# Sustained attention in dementia with Lewy bodies: a task-based fMRI study

**DOI:** 10.3389/fnagi.2026.1822185

**Published:** 2026-06-10

**Authors:** Davide Braghittoni, Greta Venturi, Lucia Guidi, Elettra Capogna, Giovanni Sighinolfi, Luca Baldelli, Giulia Amore, Chiara La Morgia, Luisa Sambati, Annalena Venneri, Raffaele Lodi, Caterina Tonon, Micaela Mitolo

**Affiliations:** 1 IRCCS Istituto delle Scienze Neurologiche di Bologna, Functional and Molecular Neuroimaging Unit, Bologna, Italy; 2 Department of Biomedical and Neuromotor Sciences (DIBINEM), University of Bologna, Bologna, Italy; 3 IRCCS Istituto delle Scienze Neurologiche di Bologna, U.O.C. Clinica Neurologica Rete Metropolitana (NeuroMet), Bologna, Italy; 4 IRCCS Istituto delle Scienze Neurologiche di Bologna, Programma di Neurogenetica, Bologna, Italy; 5 Department of Medicine and Surgery, University of Parma, Parma, Italy; 6 Department of Neuroscience, University of Sheffield, Sheffield, United Kingdom

**Keywords:** Dementia with Lewy bodies, functional MRI, precuneus, psychomotor vigilance task, sustained attention

## Abstract

**Introduction:**

Patients with Dementia with Lewy Bodies (DLB) typically experience attentional deficits. Understanding these deficits and the underlying neural correlates is crucial as sustained attention supports higher level cognitive functions. The objective of this study was to investigate the patterns of brain connectivity during a functional magnetic resonance imaging (fMRI) sustained attention task in a DLB group compared with healthy controls (HC).

**Methods:**

In this study, 30 DLB patients and 25 HC underwent a comprehensive neuropsychological assessment and a brain 3T MRI protocol that included a high-resolution structural sequence (T1-w 3D MPRAGE) and a task-based fMRI study using an auditory Psychomotor Vigilance Task (PVT).

**Results:**

DLB patients performed worse in neuropsychological tests mainly related to the visuospatial domain and had poorer performance than HC on sustained attention (i.e., PVT) in terms of lapses and longer response times. Moreover, during the PVT, the DLB group exhibited significantly lower activation in the precuneus, intracalcarine cortex, angular gyrus and cuneus, regions implicated in sustained attention.

**Discussion:**

Overall, the identification of a distinct pattern of brain activity related to attention impairment in DLB patients offers opportunities for the development of tailored cognitive stimulation treatments.

## 1 Introduction

Dementia with Lewy Bodies (DLB) is considered the second commonest form of dementia in older adults after Alzheimer’s disease (AD) (Vann Jones and O’Brien, 2014) and it is characterized by accumulation of the abnormally aggregated presynaptic protein α-synuclein (Donadio et al., 2025). In DLB, misfolded α-synuclein accumulates within neurons in the form of Lewy bodies and within neuronal structures as Lewy neurites, resulting in widespread neurodegeneration that extends beyond the brainstem to limbic and neocortical regions (McKeith et al., 2017). DLB is often clinically misdiagnosed, and only about half of the cases expected based on *post-mortem* histopathological studies are currently identified *in vivo*, suggesting substantial underdiagnosis (Arnaoutoglou et al., 2019). The cognitive profile of DLB is characterized by a progressive cognitive decline that is severe enough to interfere with daily activities, social interactions, or occupational functioning. This profile has been extensively investigated using neuropsychological batteries and these patients exhibit more pronounced attentional, executive and visuospatial impairments in the early stages of disease than patients with other forms of neurodegeneration (Wyman-Chick et al., 2024; Hemminghyth et al., 2020; McKeith et al., 2017; Metzler-Baddeley, 2007; Ferman et al., 2006). Poor performance on attention tasks in DLB is a core, often early, clinical feature reflecting fluctuations in alertness (Bradshaw et al., 2004). Cognitive fluctuations are the most elusive and least understood characteristic of DLB (Matar et al., 2020b; Cummings, 2004). Since the fluctuating nature of alertness is difficult to capture and quantify with indirect instruments (e.g., questionnaires), standardized attentional tasks may provide more objective and reliable methods for characterizing cognitive fluctuations. These tasks continuously assess cognitive performance over a period of time and allow the examination of performance variability reflecting attentional lapses (Hamilton et al., 2024; Ballard et al., 2001). The Psychomotor Vigilance Task (PVT), originally developed in 1985, may be considered an effective tool to investigate sustained attention, as it requires participants to maintain a certain level of alertness for a long period of time (Dinges and Powell, 1985). Several studies have demonstrated its sensitivity and its psychometric advantages, applicable to linguistically and culturally diverse populations (Basner and Dinges, 2011), making the PVT one of the most widely used neurobehavioral tests (Meyer et al., 2025). Changes in performance (i.e., response time) have been extensively used in sleep deprivation research. This approach also demonstrates high ecological validity, as it reflects risk in real-world performance (Basner and Dinges, 2011).

Although the PVT has been widely studied from a behavioral perspective (Tanno et al., 2025; Mason et al., 2024; Chaisilprungraung et al., 2022; Sauvet et al., 2020; Basner and Dinges, 2011), to our knowledge, studies examining its neural correlates using a task-based functional magnetic resonance imaging (fMRI) approach remain relatively limited. To date, available evidence has primarily focused on healthy individuals (Zhu et al., 2024; Pan et al., 2023; Chen et al., 2023; DiFrancesco et al., 2019; Drummond et al., 2005), with only a few investigations targeting clinical populations, including traumatic brain injury (Persson et al., 2024) and AD (La Morgia et al., 2023). None of these studies, however, focused on DLB. The present study aimed to fill this gap by investigating whether sustained attention deficits in DLB patients are associated with altered patterns of functional brain activity during a PVT task-based fMRI, through a comparison with healthy controls (HC).

Based on the prominent attentional deficits observed in DLB, we hypothesized that patients would show poorer performance on the PVT, characterized by slower reaction times and a higher number of lapses compared with HC. At the brain BOLD signal level, we expected both groups to recruit fronto-parietal networks supporting sustained attention, and reduced task-related activity in DLB patients, mainly in posterior cortical regions.

## 2 Materials and methods

### 2.1 Participants

A total of forty patients diagnosed with DLB and 25 HC, were screened between May 2022 and December 2024 prospectively at the IRCCS Institute of Neurological Sciences of Bologna, Bologna, Italy.

All patients in the DLB group had been diagnosed by an experienced neurologist (L.S.) with either probable DLB (mild stage) or probable prodromal DLB, according to the revised diagnostic criteria (McKeith et al., 2017; McKeith et al., 2020). Inclusion criteria for HC were the absence of pathological scores on the neuropsychological evaluation.

Exclusion criteria for both DLB patients and HC were as follows: (1) significant psychiatric (and neurological for HC) history, (2) presence of other severe or unstable medical illness, (3) history of alcohol or substance abuse, (4) contra-indications for MRI examination, (5) evidence of signal intensity or brain structure changes not related to DLB and (6) excessive number of omissions (> 50% of total trials) on the PVT fMRI task. In accordance with the exclusion criteria reported above, 10 DLB patients were excluded (five for incomplete MRI protocol due to low cooperation, four for a number of omissions > 50% of the total trails on the PVT fMRI task, one for normal pressure hydrocephalus).

After applying inclusion and exclusion criteria, thirty DLB (F/M: 11/19, mean age = 74.53; SD = 5.88) and 25 HC (F/M: 15/10, mean age = 69.37, SD = 6.91) were included in the analysis.

Information on medication status was collected for all DLB patients. Specifically, 10 patients (33%) were receiving both dopaminergic and cholinergic medications, 9 patients (30%) were receiving dopaminergic medication only, 3 patients (10%) were receiving cholinergic medication only, and 8 patients (27%) were receiving neither dopaminergic nor cholinergic medication. All patients were assessed in their usual medicated state, while taking their regular dopaminergic and/or cholinergic medications. For patients receiving dopaminergic treatment, the levodopa equivalent daily dose (LEDD) was calculated according to the updated conversion criteria proposed by Jost et al. (2023). Cholinergic treatment (i.e., rivastigmine) was reported as current daily dose (mg/day). Both LEDD and cholinergic daily dose values are reported in Table 1.

**TABLE 1:** Cohort characteristics, including demographic, clinical, and cognitive performance measures.

Variables/measures	DLB	HC	*P*-value (FDR)	*χ^2^*	*F*	*U*	Effect size
Demographic and clinical data							
N (females : males)	30 (11:19)	25 (15:10)	0.27	0.11			0.04
Age (years)	74.53 (5.88)	69.37 (6.91)	**0.01**		8.92		0.14
Education (years)	11.17 (4.07)	11.36 (3.53)	1.00			362	0.03
Disease duration (years)	6.69 (3.68)	NA	–	–	–	–	–
LEDD (mg/day)	416.83 (305.55)	NA	–	–	–	–	–
Cholinesterase inhibitors (mg/day)	7.05 (2.56)	NA	–	–	–	–	–
Neuropsychological test							
Mini mental state examination	25.60 (3.64)	29.32 (0.85)	**< 0.001**		23.99		0.31
Digit span forward task	5.17 (0.95)	5.80 (1.00)	0.81		4.83		0.08
Corsi block-tapping task	4.07 (1.05)	5.08 (0.81)	0.10		9.12		0.15
Rey auditory verbal learning test immediate recall	27.63 (12.41)	44.64 (9.74)	**0.01**		13.77		0.21
Rey auditory verbal learning test delayed recall	4.97 (3.47)	9.80 (3.20)	**0.02**			172	0.54
Rey-Osterrieth complex figure test delayed recall	9.53 (5.90)	19.94 (5.24)	**< 0.001**			79	0.79
Stroop test speed	35.84 (19.92)	19.26 (8.86)	0.07		9.97		0.16
Stroop test errors	4.70 (7.35)	0.24 (0.83)	0.11		8.86		0.15
Digit span backward task	3.50 (1.01)	4.04 (0.73)	1.00		3.99		0.07
Phonemic fluency test	28.30 (15.73)	39.88 (15.37)	0.96			252	0.33
Semantic fluency test	28.03 (8.77)	45.84 (10.84)	**< 0.001**			118	0.69
Rey-Osterrieth complex figure test direct copy	25.88 (9.64)	34.68 (1.99)	**0.001**		19.30		0.27
Benton judgment of line orientation	18.57 (7.86)	24.64 (2.83)	0.06			195	0.48
VOSP screening	18.93 (1.26)	19.56 (0.65)	1.00		2.39		0.04
VOSP incomplete letters	16.23 (5.08)	19.56 (0.58)	**0.04**		10.96		0.17
VOSP silhouettes	14.30 (3.69)	20.80 (3.56)	**< 0.001**		33.66		0.39
VOSP object decision	13.43 (3.05)	17.20 (1.50)	**< 0.001**		25.27		0.32
VOSP progressive silhouettes	12.87 (2.33)	9.48 (2.49)	**0.004**		16.77		0.24
VOSP dot counting	9.37 (1.33)	9.88 (0.33)	0.66			243	0.35
VOSP position discrimination	18.43 (2.65)	19.88 (0.44)	0.42		6.11		0.10
VOSP number location	7.60 (2.67)	9.44 (0.87)	0.14		8.30		0.14
VOSP cube analysis	7.20 (2.33)	9.84 (0.37)	**< 0.001**		30.04		0.36
Sleep questionnaires							
Epworth sleepiness scale	7.23 (4.44)	4.96 (3.49)	0.73		5.03		0.09
Pittsburgh sleep quality index	6.23 (3.92)	6.48 (3.94)	1.00		0.65		0.01
RBDSQ	5.60 (3.42)	1.76 (1.42)	**<0.001**		32.84		0.38
Psychomotor vigilance task (PVT)							
Omissions	5.10 (8.84)	2.12 (5.70)	0.23		4.21		0.07
Lapses	26.13 (27.12)	4.48 (5.03)	**0.002**		14.54		0.22
Median RT	432.35 (147.44)	311.92 (54.07)	**0.002**		13.97		0.21
Reciprocal RT	2.53 (0.66)	3.28 (0.53)	**< 0.001**			142	0.62

Descriptive statistics represent the means and standard deviations (SD) of raw demographic and clinical variables in DLB patients and HC. Group differences were assessed using χ^2^ test for dichotomous variables (sex only) and analysis of variance (ANOVA) or Mann–Whitney U tests, with *p*-values corrected for multiple comparisons using a false discovery rate (FDR) approach. Statistically significant results are highlighted in bold, and effect sizes are reported for each comparison. N, number of subjects; DLB, Dementia with Lewy Bodies; HC, healthy control; LEDD, levodopa equivalent daily dose; VOSP, Visual object and space perception battery; RBDSQ, REM Sleep Behavior Disorder Screening Questionnaire; RT, reaction time; NA, not applicable.

This study was approved by the local Ethical Committee (#CE 20225, Comitato Etico Area Vasta Emilia Centro) and conducted in accordance with the Declaration of Helsinki. All participants signed institutional review board–approved consent forms.

### 2.2 Neuropsychological assessment

All participants underwent an extensive neuropsychological assessment, performed on a different day than the fMRI protocol to avoid excessive cognitive fatigue, within an interval of a maximum 3 weeks from the MRI examination. The neuropsychological assessment included a measure of global cognition (i.e., the Mini-Mental State Examination, MMSE; Magni et al., 1996) and a comprehensive battery assessing a wide range of cognitive abilities: short- and long-term verbal and visuo-spatial memory (Digit Span Forward Task; Corsi Block-Tapping Task; Rey Auditory Verbal Learning Test immediate and delayed recall; Rey-Osterrieth Complex Figure Test delayed recall) (Monaco et al., 2013; Carlesimo et al., 1996; Caffarra et al., 2002b), executive functions (Stroop Test; Digit Span Backward Task) (Caffarra et al., 2002a; Monaco et al., 2013), verbal fluency (Phonemic and Semantic Fluency Test) (Carlesimo et al., 1996; Zarino et al., 2014), visuoperceptual/visuospatial functions (Benton Judgment of Line Orientation Test; Rey-Osterrieth Complex Figure Test direct copy and Visual Object and Space Perception Battery, VOSP) (Ferracuti et al., 2000; Caffarra et al., 2002b; Warrington and James, 1991). In addition, three questionnaires were administrated to assess sleep quality and sleep behavior: the Epworth Sleepiness Scale (ESS) (Vignatelli et al., 2003), Pittsburgh Sleep Quality Index (PSQI) (Curcio et al., 2013) and the REM Sleep Behavior Disorder Screening Questionnaire (RBDSQ) (Marelli et al., 2016).

### 2.3 Brain MRI acquisition

The standardized brain MRI protocol was performed using a high-field 3 T MRI scanner (Siemens MAGNETOM Skyra) equipped with a head–neck high-density (64 channels) array coil. The MRI protocol included a T1-weighted 3D Magnetization-Prepared Rapid Gradient-Echo Imaging sequence (MPRAGE, 176 continuous sagittal slices, 1-mm isotropic voxel, no slice gap, echo time (TE) = 2.98 ms, repetition time (TR) = 2,300 ms, Inversion Time (IT) = 900 ms, flip angle = 9°, acquisition matrix = 256 × 256, pixel bandwidth = 240 Hz, in-plane acceleration factor = 2, duration ∼5 min), a T2-weighted 3D fluid-attenuated inversion recovery (FLAIR) sequence (SPACE, 176 sagittal acquisition slices, 1-mm isotropic voxel, no slice gap, TE = 428 ms, TR = 5,000 ms, IT = 1,800 ms, flip angle = 120°, acquisition matrix = 256 × 256, pixel bandwidth = 780 Hz, in-plane acceleration factor = 2, duration ∼5 min) and a task-based fMRI sequence (Gradient Echo—Echo Planar Imaging, GRE-EPI, isotropic voxel 2.5-mm, FOV 235 mm, repetition time TR = 735 ms, echo time TE = 37 ms, flip angle 53°, duration ∼ 8 min).

#### 2.3.1 Structural MRI preprocessing

The 3D T1-weighted images were processed using the standard FreeSurfer pipeline (recon-all, version 6.0), which includes automated segmentation of cortical and subcortical structures and cortical parcellation of the left and right hemispheres based on the Desikan-Killiany atlas (Desikan et al., 2006). Volumes of the various brain regions were computed for morphometric analysis.

### 2.4 PVT-fMRI task and behavioral metrics

The auditory PVT used in the current study was similar to the version developed by Drummond et al. (2005) and consisted in a simple measure of reaction time (RT) to repetitive stimuli. The task involved the presentation of a series of sounds (duration = 0.5 s) implemented in E-prime 3.0 (Psychology Software Tools, United States) and administered via MR-compatible headphones. Each stimulus was separated by random silent interstimulus intervals ranging between 1 and 9 s for an overall duration of ∼8 min and a total number of trials equal to 95. The participant had to respond to each sound as quickly as possible by pressing a button on an MR-compatible handgrip (Response Grip, NordicNeuroLab AS, Bergen, Norway). A response was considered valid if the RT ≥ 100 ms (Basner and Dinges, 2011). The specific PVT performance metrics, used in our analysis, included omissions (no answer), number of lapses (defined as RTs ≥ 500 ms), median RT and mean 1/RT (also called reciprocal RT) (Basner and Dinges, 2011; Lim and Dinges, 2008). Before the fMRI session, all participants were trained by an expert neuropsychologist (L.G. or D.B.) to ensure proper execution of the PVT task (task accuracy > 70%) in the scanner.

#### 2.4.1 Functional MRI preprocessing

Task-based fMRI data analyses were conducted using FSL.^1^ As previously described (Muccioli et al., 2023; Evangelisti et al., 2021), pre-processing included motion correction using MCFLIRT, slice timing correction to adjust for acquisition differences, high-pass filtering with a cut-off of 60 s to remove low-frequency signal drifts, and spatial smoothing with a 5 mm FWHM Gaussian kernel to enhance the signal-to-noise ratio. In correspondence of each auditory stimulus a rectangular function of duration 0.735 s (equal to 1 TR, i.e., the temporal resolution of the fMRI sequence) was defined and convolved with a double-gamma hemodynamic response function. This model is hypothesized to capture the blood-oxygenation-level-dependent (BOLD) signal response following the auditory stimulus, reflecting the patient’s brain activity while sustaining attention to perform the task. The single-subject contrast was defined as the parameter estimate associated with this explanatory variable. The fMRI data were fit voxelwise to the defined model design, at the single-subject level, using a General Linear Model (GLM) (Woolrich et al., 2001). To minimize the influence of movement-related artifacts, motion parameters extracted from the pre-processing stage were incorporated as nuisance covariates. Following single-subject analyses, the contrasts of parameter estimates were normalized to the standard MNI space by registering functional images to each individual’s T1-weighted image using FSL *epi_reg*, followed by non-linear normalization to MNI space using FSL FNIRT, allowing for the subsequent group-level comparisons and statistical evaluations, using the FLAME1 (FMRIB’s Local Analysis of Mixed Effects) model. Additionally, log files were recorded during data acquisition to extract subject response metrics, enabling further assessment of RT and performance accuracy.

### 2.5 Statistical analysis

Descriptive statistics are reported as means and standard deviations (SD) for raw demographic and clinical variables for DLB patients and HC. Group differences in dichotomous variables (sex only) were assessed using the chi-square (χ^2^) test. Raw neuropsychological data were adjusted for the effects of sex, age, and education, while volumetric data were corrected for sex, age, and estimated total intracranial volume (eTIV) using the residual method. Specifically, a multivariate linear regression model between each variable of interest and the nuisance variables was performed within the control group, and the estimated parameters were used to correct the raw values for every participant. The Shapiro-Wilk test was then applied to the residuals of each participant to assess normality. In case of two-group comparisons, either an analysis of variance (ANOVA) or a Mann-Whitney U test was used to test for group differences, based on the normality of the data distribution. For each comparison, the corresponding test statistic (F value for ANOVA or U value for Mann–Whitney U) and *p*-value are reported. Associations between significant volumetric measures and PVT performance or neuropsychological data were tested using Spearman partial correlations adjusted for age, sex, education, and eTIV. Additionally, false discovery rate (FDR) correction was applied to multivariate models. Statistical significance was set at adjusted *p* < 0.05. *P*-values were further classified as significant (*p* < 0.05), highly significant (*p* < 0.01), and extremely significant (*p* < 0.001).

Effect size for group differences was assessed using Cohen’s d for continuous variables and Cramér’s V for dichotomous variables. For non-parametric Mann–Whitney U tests, effect size r was calculated as r = | Z| /√N, where Z is the standardized U statistic and N is the total sample size. The statistical analysis was conducted using an in-house script developed in Python 3.11.

Group-level fMRI analyses were performed using FMRIB’s Local Analysis of Mixed Effects (FLAME1) with an unpaired two-group difference. Sex, age, and chronic white matter lesion load—estimated with the Lesion Segmentation Tool (LST)^2^ —were included as covariates to control for potential confounding factors. Statistical significance was determined using cluster-based correction (Z > 3.1, cluster-wise *p* < 0.05) as implemented in FSL FEAT. Significant clusters were separated based on the Harvard-Oxford cortical and subcortical atlas^3^ (Makris et al., 2006; Frazier et al., 2005; Desikan et al., 2006; Goldstein et al., 2007), and only regions with more than 20 significant voxels were retained to exclude very small clusters and improve interpretability, without influencing statistical inference (Roiser et al., 2016; Woo et al., 2014). Associations between significant activation areas and PVT performance or neuropsychological data were tested using Spearman partial correlations adjusted for age, sex, and education, and corrected with FDR. Sensitivity analyses were performed to assess potential group × covariate interaction effects (age, sex, and white matter lesion load) on significant activation areas. Activation values were entered into separate linear models including group, the covariate of interest, and the corresponding interaction term.

To assess the potential influence of pharmacological treatment, additional analyses were performed within the DLB group. Specifically, contrast of parameter estimates (COPE) values derived from the first-level GLM were used as an index of overall task-related activity. COPE values and PVT performance metrics were compared between patients receiving *vs* not receiving dopaminergic and cholinergic treatments. In addition, partial correlations were performed between both COPE values and PVT measures and medication status, considering each treatment separately while controlling for the presence of the other form of treatment.

## 3 Results

### 3.1 Group differences in neuropsychological tests and PVT behavioral data

Demographic and neuropsychological data are presented in Table 1. DLB and HC groups did not differ in terms of sex and education (*p* > 0.05), whereas a significant difference was found in age (DLB patients were older than HC). For this reason, age was included as a covariate in all analyses. The mean interval between neuropsychological assessment and MRI examination was 18.33 days. As expected, DLB patients showed poorer performance on neuropsychological tests than HC, in particular they obtained worse scores in global cognition (MMSE, *p* < 0.001), in Object and Spatial perception (VOSP Silhouettes, *p* < 0.001; VOSP Cube Analysis, *p* < 0.001; VOSP Object Decision, *p* < 0.001; VOSP Progressive Silhouettes, *p* = 0.004; VOSP Incomplete Letters, *p* = 0.04), visuo-spatial long-term memory (Rey-Osterrieth Complex Figure Test delayed recall, *p* < 0.001) and constructional praxis (Rey-Osterrieth Complex Figure Test direct copy, *p* = 0.001). Furthermore, patients showed significantly worse performance than controls in verbal fluency (Semantic Fluency Test, *p* < 0.001), verbal memory (Rey Auditory Verbal Learning Test immediate recall, *p* = 0.01 and delayed recall, *p* = 0.02) and in the RBDSQ, *p* < 0.001 (see Supplementary Figure 2A). On the PVT (behavioral data only), DLB patients performed worse than HC in terms of reciprocal RT, *p* < 0.001, number of lapses (*p* = 0.002) and median RT (*p* = 0.002) (see Supplementary Figure 2B).

No significant group × covariate interaction effects were observed across significant activation areas (see Supplementary Figure 1).

### 3.2 Brain volumetry differences

All brain MRI images of DLB and HC were assessed by experienced neuroradiologist and were of good quality. Structural analysis revealed that the DLB group, when compared with HC, showed volumetric loss in different temporo-parietal, occipital and orbitofrontal areas (e.g., left superior temporal sulcus cortex, left inferior parietal cortex, left middle temporal gyrus, right inferior parietal lobule, left entorhinal cortex, left fusiform gyrus, right superior temporal gyrus, right lateral occipital cortex, bilateral lateral orbitofrontal cortex) and subcortical structures (e.g., bilateral hippocampus and bilateral amygdala). See Supplementary Table 1 and Supplementary Figure 3 for an overview of all volumetric results.

No significant associations emerged between regional volumetric measures and PVT performance. Instead, at an uncorrected statistical threshold several significant associations were found. Specifically, activation in the left inferior parietal cortex was associated with performance on the VOSP Object Decision subtest (*r* = 0.575, *p* = 0.002). Right inferior parietal cortex was associated with the VOSP Object Decision subtest (*r* = 0.481, *p* = 0.013) and the VOSP Incomplete Letters subtest (*r* = 0.464, *p* = 0.017). Left lateral orbitofrontal cortex was associated with Semantic Fluency Test (*r* = 0.545, *p* = 0.004), Rey Auditory Verbal Learning Test delayed recall (*r* = 0.523, *p* = 0.006), Rey-Osterrieth Complex Figure Test delayed recall (*r* = 0.491, *p* = 0.011), Rey Auditory Verbal Learning Test immediate recall (*r* = 0.434, *p* = 0.027), the VOSP Object Decision subtest (*r* = 0.411, *p* = 0.037), and Rey-Osterrieth Complex Figure Test direct copy (*r* = 0.402, *p* = 0.042). Right amygdala was associated with the VOSP Progressive Silhouettes subtest (*r* = 0.509, *p* = 0.008), Rey-Osterrieth Complex Figure Test direct copy (*r* = 0.460, *p* = 0.018), and Rey-Osterrieth Complex Figure Test delayed recall (*r* = 0.433, *p* = 0.027). Right hippocampus was associated with Semantic Fluency Test (*r* = 0.506, *p* = 0.008), the VOSP Incomplete Letters subtest (*r* = 0.472, *p* = 0.015), and Rey-Osterrieth Complex Figure Test direct copy (*r* = 0.446, *p* = 0.022). Left hippocampus was associated with Semantic Fluency Test (*r* = 0.502, *p* = 0.009), Rey-Osterrieth Complex Figure Test direct copy (*r* = 0.474, *p* = 0.014), the VOSP Silhouettes subtest (*r* = 0.460, *p* = 0.018), and the VOSP Incomplete Letters subtest (*r* = 0.458, *p* = 0.019). Right isthmus cingulate was associated with Rey Auditory Verbal Learning Test delayed recall (*r* = 0.417, *p* = 0.034). Left pericalcarine cortex was associated with the VOSP Cube subtest (*r* = 0.409, *p* = 0.038). See Supplementary Table 2 for an overview of the correlations between regional volumetric measures and neuropsychological performance in the DLB group.

### 3.3 Brain BOLD signal during the PVT-fMRI task

**FIGURE 1:**
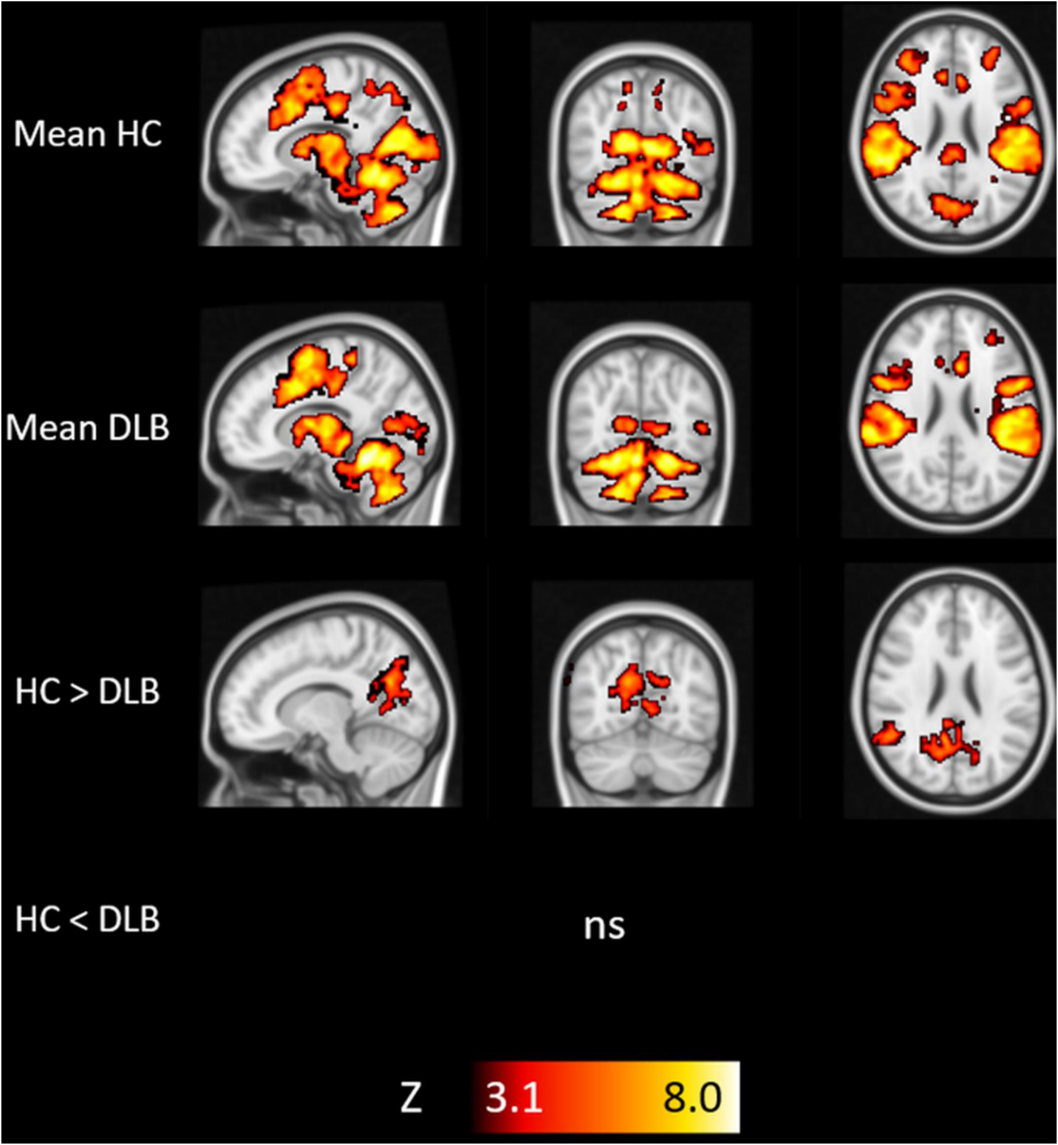
Average task-related functional MRI activation maps during PVT in HC and DLB groups and differences between HC and DLB. Representation on the MNI space (2 mm resolution) of the significant activation areas. First row: clusters represent the average significant activations in the HC group. Second row clusters show average activations in the DLB group. Third row: regions of significantly higher activation in HC than in DLB during the PVT-fMRI task. Fourth row: regions of significantly lower activation in HC than in DLB during the PVT-fMRI task. Images are shown in sagittal, coronal and axial views. Color scale represents the Z-statistic value. PVT, Psychomotor vigilance task; HC, healthy control; DLB, dementia with Lewy body; ns, not significant.

The brain activation maps during the PVT-fMRI task are shown in Figure 1. In both groups, the PVT-induced BOLD signal was distributed in cortical and subcortical regions that are mainly involved in sustained attention, while also encompassing areas associated with motor responses and auditory processing. We found significant clusters of increase in BOLD signal in frontoparietal region (e.g., bilateral precentral gyrus, bilateral premotor cortex, bilateral central opercular cortex, left postcentral gyrus, right supramarginal gyrus posterior division and left supramarginal gyrus anterior division), temporal (bilateral superior temporal gyrus posterior division, bilateral planum temporale and bilateral Heschl’s gyrus) and occipital regions (e.g., bilateral lingual gyrus, bilateral intracalcarine cortex, bilateral occipital pole region). Subcortical clusters overlapped with regions including the bilateral thalamus and putamen. In addition, there were some clusters of significant activations in the cerebellum (i.e., bilateral lobule V, bilateral lobule VI). A comparison between the two groups showed significantly lower BOLD signal in DLB patients in clusters overlapping with the precuneus, intracalcarine cortex, angular gyrus, lingual gyrus and cuneus. No significant associations were observed between PVT performance measures and task-related BOLD signal after correction for multiple comparisons. However, at an uncorrected statistical threshold, the number of omissions during the PVT was associated with activation in the lingual gyrus in both the DLB group (*r* = 0.381, *p* = 0.050, uncorrected) and the HC group (*r* = 0.471, *p* = 0.027, uncorrected). As these associations did not survive FDR correction, they should be considered only as exploratory. Statistically significant clusters, along with their voxel counts, are reported in Table 2 and are visually represented in Figure 1. In addition, no significant effects of dopaminergic or cholinergic treatment were observed on either COPE values or PVT performance metrics, and no associations emerged between medication status and either neural or behavioral measures.

**TABLE 2:** Differences between DLB patients and HC (DLB < HC) in activation maps during the PVT-fMRI task.

Brain region	Total voxel (N)	Left voxel (N)	Right voxel (N)
Precuneus	788	145	643
Intracalcarine cortex	277	167	110
Angular gyrus	248	0	248
Lingual gyrus	93	65	28
Cuneus	78	5	73
Cingulate gyrus, posterior division	67	35	32
Supramarginal gyrus, posterior division	58	0	58
Supracalcarine cortex	52	5	47
Heschl’s gyrus	49	0	49
Superior temporal gyrus, posterior division	47	0	47
Planum temporale	35	0	35
Lateral occipital cortex, superior division	33	0	33
Occipital pole	20	20	0

The number of voxels (total and lateralized) per brain region showing significantly lower activation in DLB than in HC (*p* < 0.001) is reported. Anatomical labels correspond to the Harvard–Oxford cortical and subcortical atlas, together with the voxel count for each region. N indicates the number of voxels.

## 4 Discussion

The original contribution of this study was to detect for the first time the pattern of brain activity during the PVT fMRI task in a group of patients with Dementia with Lewy bodies.

Cerebral responses to the PVT in both groups involved cortical and subcortical brain regions associated with sustained attention, localized in the frontal, parietal, and occipital lobes. Subcortical regions included the bilateral thalamus and putamen, with additional increase in BOLD signal observed in the cerebellum (Chen et al., 2023; Drummond et al., 2005; Lawrence et al., 2003). In addition, the precentral gyrus and supplementary motor area are regions typically associated with motor function (DiFrancesco et al., 2019; Halsband et al., 1993), whereas the posterior division of the middle temporal gyrus, the planum temporale and Heschl’s gyrus are associated with auditory processing (Warrier et al., 2009).

However, during PVT performance, DLB patients exhibited decrease in BOLD signal in clusters primarily overlapping with the precuneus, followed by the intracalcarine cortex, angular gyrus, lingual gyrus, and cuneus. Given that DLB patients were compared with a HC group who completed the same task, and that no significant between-group differences emerged in regions typically associated with motor or auditory processing, it is reasonable to interpret the lower BOLD signal observed in these posterior regions as more likely reflective of poorer PVT performance by DLB patients relative to healthy controls.

The precuneus is one of the most highly connected hub regions in the brain, involved in the default mode network (DMN) and in the para-cingulate network, which is a subnetwork of the larger central executive network (CEN) (Dadario and Sughrue, 2023). For this reason, this structure is considered as a key point of vulnerability that, when structurally damaged and/or functionally disrupted, is related to numerous neurodegenerative diseases (Dadario and Sughrue, 2023). Although evidence has implicated multiple neuronal networks in the mediation of sustained attention (Lawrence et al., 2003; Langner and Eickhoff, 2013), the frontal and parietal networks appear as the most relevant to sustained attention.

The clusters overlapping with the precuneus are consistent with previous evidence indicating a central role of this region in sustained attention, as recently demonstrated using the PVT-fMRI task in healthy young individuals (Chen et al., 2023). The authors examined whether acute physical exercise (Tai Chi Chuan) was effective in enhancing sustained attention and performance-related brain activation among young adults. They found that a single session of acute physical exercise significantly improved sustained attention, as evidenced by shorter reaction times during the PVT and greater activation in the cuneus and precuneus after exercise compared with the resting condition. Greater task-evoked activation is suggestive of better overall sustained attention, indicating improved ability to allocate attentional resources over time. The precuneus has been identified as a possible hub functionally connected with the Dorsal Attention Network (DAN) during sustained attention tasks (Li et al., 2020), thus alteration of this area might result in poorer performance in attentional tasks. Moreover, growing evidence further supports, through applied task-based fMRI (Lyu et al., 2021) and lesion symptom mapping (Yeager et al., 2022), the involvement of the central precuneus within a broader network implicated in cognitive control and executive functions.

In line with this evidence, the cuneus and precuneus seem to be a vulnerable hub in the prodromal stage of α-synucleinopathies, as confirmed in a recent study on idiopathic REM sleep behavior disorder (iRBD) with mild cognitive impairment (Mattioli et al., 2021). These authors reported relative hypometabolism assessed by the [^18^]F-FDG-PET technique in iRBD with mild cognitive impairment in the cuneus and precuneus, that may reflect neural dysfunction in posterior cortical areas that is already detectable in the prodromal stage, as the synucleinopathy progresses. Furthermore, the clusters overlapping with the intracalcarine cortex and inferior parietal lobe (supramarginal gyrus and angular gyrus)—which showed significantly lower BOLD signal in our DLB patients than in HC—also are in line with previous evidence supporting the involvement of these regions in sustained attention and encoding salient environmental events (Nakahara et al., 2023; Singh-Curry and Husain, 2009). Moreover, although the associations did not survive FDR correction and should therefore be interpreted cautiously, the observed relationship between greater lingual gyrus activation and a higher number of omissions during the PVT in both the DLB and HC groups may plausibly suggest that engagement of internally oriented, imagery-related processes (Benedek et al., 2016) interferes with sustained external attention.

As for the functional architecture of the brain, there is only limited evidence related to task-based fMRI in dementia patients due to the complexity and the severity of the cognitive profile (Matar et al., 2020a). In DLB patients only a few studies (Firbank et al., 2018; Kobeleva et al., 2017; Firbank et al., 2016; Taylor et al., 2012; Sauer et al., 2006) have used task-based fMRI. The evidence, therefore, is limited, while the majority of the studies of patients with DLB have used resting state fMRI (Kucikova et al., 2024; Tang et al., 2022). This study, given the prominent attentional impairments in DLB, used the PVT fMRI-task and showed that this simple paradigm, specifically designed to assess sustained attention (Dinges and Powell, 1985), is also feasible in patients with DLB, supporting previous research involving individuals with AD (La Morgia et al., 2023). These authors showed lower levels of response in AD than controls within occipital, temporal, insular, parietal, and cerebellar cortices, whereas DLB patients showed significantly lower activation mainly in parietal cortex (precuneus and angular gyrus) and occipital cortex (intracalcarine cortex, lingual gyrus and cuneus). While direct comparisons should be interpreted with caution, these findings may indicate that attentional impairments in patients with AD and DLB are associated with partially distinct patterns of network involvement.

Indeed, our results showed that the PVT is highly sensitive in detecting functional modulation within the precuneus hub in DLB patients, providing a potential basis for rehabilitative interventions. Sustained attention represents the foundational level within the hierarchical theory of attention (Sohlberg and Mateer, 2001). Therefore, neuropsychological rehabilitation strategies targeting this domain may yield generalized benefits across higher-order attentional components and complex cognitive functions.

In this perspective, the PVT task-based fMRI could be useful for exploring functional connectivity before and after attention training in DLB, as proposed in a pilot study on patients with traumatic brain injury (Persson et al., 2024). Moreover, recent work has suggested that non-invasive brain stimulation techniques, such as transcranial magnetic stimulation (TMS), have been successful in treating multiple neurocognitive disorders by modulating activity in brain networks (Koch et al., 2022; Weiler et al., 2020; Canter et al., 2016). Within this framework, the PVT task-based fMRI paradigm could be a useful tool for exploring patterns of functional connectivity before and after neuromodulation.

The PVT has been used for the last 30 years as a sensitive tool for measuring degradation of sustained attention performance under sleep deprivation with high test-retest reliability and low practice effects (Basner and Dinges, 2011; DiFrancesco et al., 2019). Metrics obtained from the PVT, such as lapses, have been shown to differentiate successfully individuals with dementia (i.e., frontotemporal lobar degeneration spectrum and Alzheimer’s disease) from healthy individuals and to be a test to screen and identify rapidly individuals in need of further cognitive examination (Tanno et al., 2025; Manuel et al., 2019).

In our study, from a behavioral perspective, DLB patients showed slower RTs than HC, as shown by longer median reaction time and reciprocal reaction time, suggesting a delayed processing speed in DLB, possibly reflecting slower cognitive processing speed in these patients (Firbank et al., 2018). Moreover, DLB showed also greater lapses and variability in performance, consistent with a previous study in this population with a continuous performance task (CPT) that also involves sustained attention (Hamilton et al., 2024), indicating failure of monitoring processes and potentially fluctuation in alertness.

For volumetric data, the DLB group showed greater gray matter atrophy in temporal, parietal, occipital and orbitofrontal areas, consistent with previous evidence (Nedelska et al., 2015; Watson et al., 2012; Ballmaier et al., 2004). However, in the absence of significant associations between behavioral performance on the PVT task and regional atrophy, the observed deficits may not be fully explained by structural atrophy alone, but may instead reflect functional network impairment. In detail, poorer PVT performance observed in DLB patients during the task-based fMRI paradigm appears more likely to reflect dysfunction of large-scale functional networks supporting sustained attention, rather than being directly explained by regional atrophy alone. Furthermore, it is noteworthy that the regions showing lower BOLD signal in the DLB group were not fully overlapping with those showing the greatest degree of atrophy, which were predominantly located in the temporal lobe. This interpretation is consistent with previous evidence in DLB showing that functional connectivity changes may be more closely related to clinical symptoms than gray matter loss by itself (Kucikova et al., 2024; Chabran et al., 2020).

Conversely, in our DLB group, several associations emerged at an uncorrected statistical threshold. Poorer performance relative to HC in object and spatial perception (i.e., VOSP Progressive Silhouettes, VOSP Incomplete Letters, and VOSP Silhouettes) appeared to be related to greater temporal and parietal cortical atrophy; poorer visuo-spatial long-term memory (i.e., Rey–Osterrieth Complex Figure Test delayed recall) to greater lateral orbitofrontal and temporal cortical atrophy; poorer constructional praxis (i.e., Rey–Osterrieth Complex Figure Test direct copy) and semantic verbal fluency to greater temporal and lateral orbitofrontal cortical atrophy; and poorer verbal memory (i.e., Rey Auditory Verbal Learning Test immediate and delayed recall) to greater lateral orbitofrontal cortical atrophy.

The pattern of cognitive impairments detected in global cognition (Mini-Mental State Examination) and in specific domains by our DLB sample is similar to the pattern of impairments typically observed in this disorder (Devenyi and Hamedani, 2024; McKeith et al., 1996). This study, however, present some limitations, for instance the relatively small sample size may reduce the generalizability of the results. Additionally, cognitive fluctuations were not assessed using *ad hoc* scales, but only clinically observed or reported by caregivers. The absence of a clinical control group (e.g., patients with AD or any other type of neurodegenerative disorder) limits inference on the specificity of the observed fMRI results. Nevertheless, indirect comparisons can be made with results reported in previous studies, particularly by La Morgia et al. (2023), in which patients with AD showed less activation in some areas than HC during the PVT-fMRI task that did not fully overlap with those identified in the present study. A further limitation concerns the potential influence of medication status on both behavioral performance and BOLD responses. In detail, dopaminergic and cholinergic treatments are known to modulate attentional processes and their underlying neural networks (Decker and Duncan, 2020; Firbank et al., 2018; Tahmasian et al., 2015). Although our additional analyses revealed no significant effects of dopaminergic or cholinergic treatment, a residual influence of pharmacological treatment on both neural and behavioral measures cannot be entirely discarded. The absence of a control condition in the fMRI paradigm should also be acknowledged as a limitation. Although the observed activation pattern was consistent with regions commonly implicated in sustained attention, the lack of an explicit control task prevents a fully specific attribution of these effects, as motor responses related to button pressing and auditory/sensory processing of the stimuli may also have contributed to the observed pattern of BOLD signal. Finally, lesion load showed a non-normal (right-skewed) distribution. However, as this variable was included only as a covariate of no interest, it is unlikely to have influenced the main results. Future studies may nonetheless explore alternative modeling approaches or transformations.

Overall, the present findings should be interpreted with caution given the preliminary nature of the study. Nevertheless, the identification of these alterations is noteworthy and supports the relevance of the proposed approach. Based on these results, longitudinal investigations are warranted to establish their temporal stability and their utility in monitoring disease progression.

## 5 Conclusion

During PVT performance, DLB patients showed longer reaction times, along with increased response variability and a higher number of lapses compared with healthy controls, indicating deficits in sustained attention. Functional MRI during the PVT revealed lower BOLD signal in the DLB group relative to controls, primarily in clusters overlapping with the precuneus, intracalcarine cortex, angular gyrus, and cuneus—critical regions for sustained attention. Characterizing these patterns of cortical dysfunction in DLB may enhance *in vivo* diagnostic accuracy and support the development of more targeted rehabilitation strategies.

## Data Availability

The raw data supporting the conclusions of this article will be made available by the authors, without undue reservation.

## Footnotes

1
https://fsl.fmrib.ox.ac.uk/fsl/

2
https://www.applied-statistics.de/lst.html

3
https://neurovault.org/collections/262/
