# Supplementary material for: Sustained attention in dementia with Lewy bodies: a task-based fMRI study
Source: Front Aging Neurosci. 2026 Jun 10;18:1822185. doi: 10.3389/fnagi.2026.1822185 (PMC13290862; doi:10.3389/fnagi.2026.1822185)
Supplement: Supplementary file 1 [file Data_Sheet_1.docx]

**Supplementary Table 1. Brain volumetry in DLB patients compared with HC.** Cortical and subcortical regions showing significant volume differences between patients with dementia with Lewy bodies (DLB) and healthy controls (HC). Group differences were assessed using analysis of variance (ANOVA) or Mann–Whitney U tests, with p values corrected for multiple comparisons using false discovery rate (FDR). Statistically significant results are indicated (*p < 0.05; **p < 0.01). Effect sizes are reported for each region. DLB = Dementia with Lewy Bodies; HC = healthy controls; R = right; L = left; bankssts = Banks of the Superior Temporal Sulcus.

| **Anatomical region** | ***p value*** | ***significance*** | ***F*** | ***U*** | ***Effect size*** |
| --- | --- | --- | --- | --- | --- |
| L bankssts | 0.002 | ** | 20.84 |  | 0.29 |
| L inferior parietal | 0.002 | ** | 19.41 |  | 0.28 |
| L hippocampus | 0.003 | ** | 12.66 |  | 0.20 |
| L amygdala | 0.003 | ** |  | 576 | -0.54 |
| R hippocampus | 0.003 | ** | 13.53 |  | 0.20 |
| R amygdala | 0.003 | ** | 15.97 |  | 0.25 |
| L lateral orbitofrontal | 0.007 | ** | 13.69 |  | 0.21 |
| L middle temporal | 0.007 | ** | 14.69 |  | 0.22 |
| R lateral orbitofrontal | 0.007 | ** | 14.08 |  | 0.21 |
| R inferior parietal | 0.008 | ** | 13.09 |  | 0.20 |
| L entorhinal | 0.008 | ** | 12.07 |  | 0.19 |
| L fusiform | 0.008 | ** | 12.39 |  | 0.20 |
| R superior temporal | 0.008 | ** | 11.99 |  | 0.18 |
| L pars triangularis | 0.010 | ** | 11.34 |  | 0.18 |
| R isthmus cingulate | 0.014 | * | 10.17 |  | 0.17 |
| R lateral occipital | 0.014 | * | 10.26 |  | 0.17 |
| L pericalcarine | 0.017 | * | 9.50 |  | 0.15 |
| L rostral middle frontal | 0.017 | * | 9.34 |  | 0.15 |
| R middle temporal | 0.018 | * | 9.03 |  | 0.15 |
| L isthmus cingulate | 0.028 | * | 7.99 |  | 0.13 |
| R fusiform | 0.028 | * | 7.87 |  | 0.13 |
| L superior temporal | 0.035 | * | 7.29 |  | 0.12 |
| R pericalcarine | 0.045 | * | 6.72 |  | 0.12 |

**Supplementary Table 2. Correlations between regional volumetric measures and neuropsychological performance in the DLB group.** Spearman partial correlations between neuropsychological test scores and regional volumetric measures that showed significant group differences (DLB vs HC) are reported for the DLB group. Analyses were adjusted for age, sex, education, and estimated total intracranial volume (eTIV). Only associations with an uncorrected p-value < 0.05 are shown. Both uncorrected and corrected p-values are reported. DLB = Dementia with Lewy Bodies; HC = healthy controls; R = right; L = left; VOSP = Visual Object and Space Perception Battery; VOSP Silh. = VOSP Silhouettes; VOSP Cube = VOSP Cube Analysis; VOSP Obj. Decision = VOSP Object Decision; VOSP Progr. Silh. = VOSP Progressive Silhouettes; VOSP Inc. Letters = VOSP Incomplete Letters; Figure Recall = Rey-Osterrieth Complex Figure Test delayed recall; Semantic Fluency = Verbal Fluency Test; Figure Copy = Rey-Osterrieth Complex Figure Test direct copy; RAVLT Immediate = Rey Auditory Verbal Learning Test immediate recall; RAVLT Delayed = Rey Auditory Verbal Learning Test delayed recall.

| **Anatomical region** | **Neuropsychological test** | ***r*** | ***Uncorrected P value*** | ***Corrected***  ***P value*** |
| --- | --- | --- | --- | --- |
| L inferior parietal | VOSP Obj. Decision | 0.575 | 0.002 | 0.317 |
| L lateral orbitofrontal | Semantic Fluency | 0.545 | 0.004 | 0.317 |
| L lateral orbitofrontal | RAVLT Delayed | 0.523 | 0.006 | 0.317 |
| R amygdala | VOSP Progr. Silh. | 0.509 | 0.008 | 0.317 |
| R hippocampus | Semantic Fluency | 0.506 | 0.008 | 0.317 |
| L hippocampus | Semantic Fluency | 0.502 | 0.009 | 0.317 |
| L lateral orbitofrontal | Figure Recall | 0.491 | 0.011 | 0.317 |
| R inferior parietal | VOSP Obj. Decision | 0.481 | 0.013 | 0.317 |
| L hippocampus | Figure Copy | 0.474 | 0.014 | 0.317 |
| R hippocampus | VOSP Inc. Letters | 0.472 | 0.015 | 0.317 |
| R inferior parietal | VOSP Inc. Letters | 0.464 | 0.017 | 0.317 |
| L hippocampus | VOSP Silh | 0.460 | 0.018 | 0.317 |
| R amygdala | Figure Copy | 0.460 | 0.018 | 0.317 |
| L hippocampus | VOSP Inc. Letters | 0.458 | 0.019 | 0.317 |
| R hippocampus | Figure Copy | 0.446 | 0.022 | 0.350 |
| L lateral orbitofrontal | RAVLT Immediate | 0.434 | 0.027 | 0.360 |
| R amygdala | Figure Recall | 0.433 | 0.027 | 0.360 |
| R isthmus cingulate | RAVLT Delayed | 0.417 | 0.034 | 0.428 |
| L lateral orbitofrontal | VOSP Obj. Decision | 0.411 | 0.037 | 0.435 |
| L pericalcarine | VOSP Cube | 0.409 | 0.038 | 0.435 |
| L lateral orbitofrontal | Figure Copy | 0.402 | 0.042 | 0.442 |


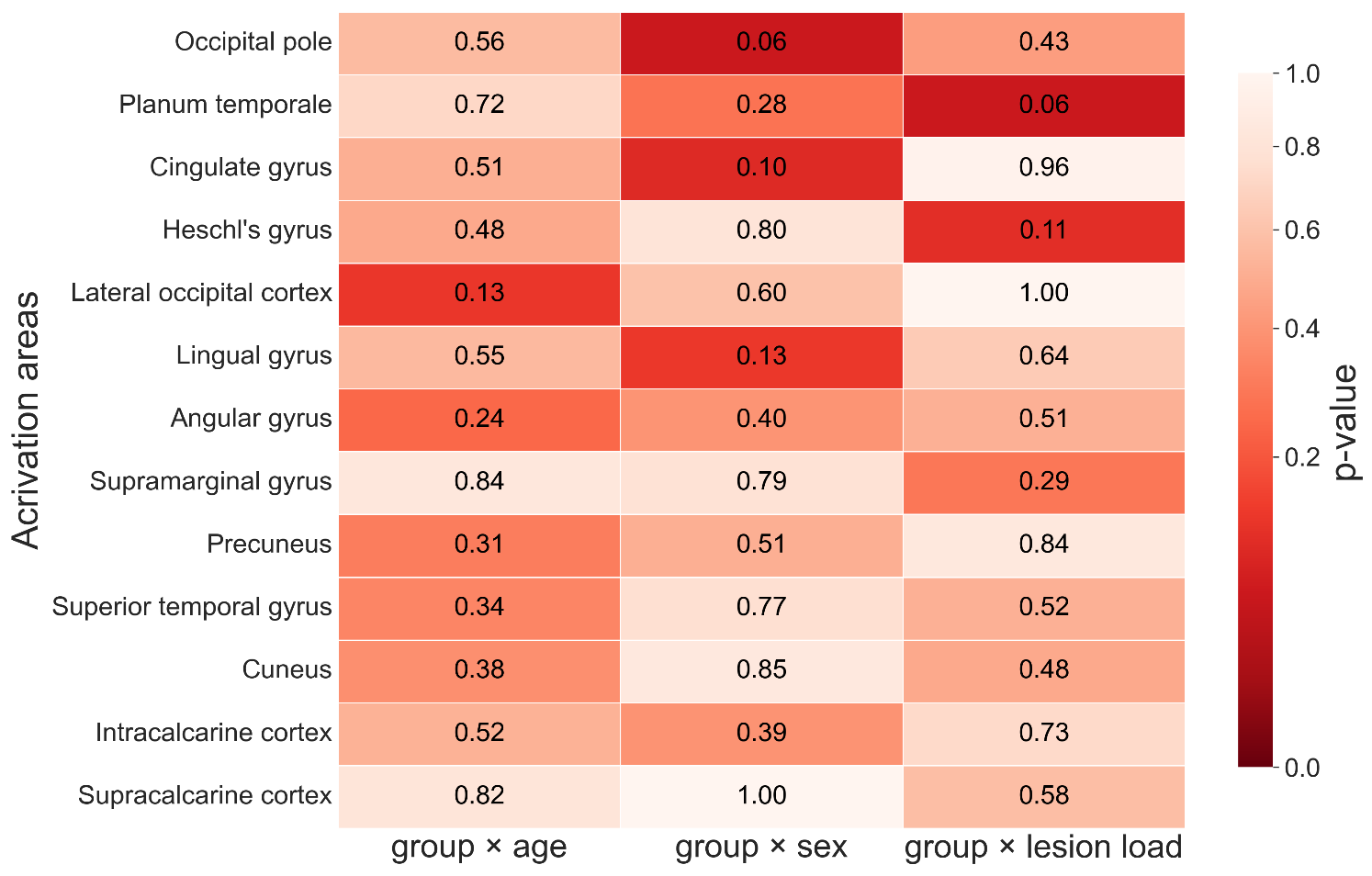


**Supplementary Figure 1. Interaction effects across BOLD signal clusters.** Heatmap of corrected p-values for group × covariate interaction effects (age, sex, and white matter lesion load) across significant activation areas.


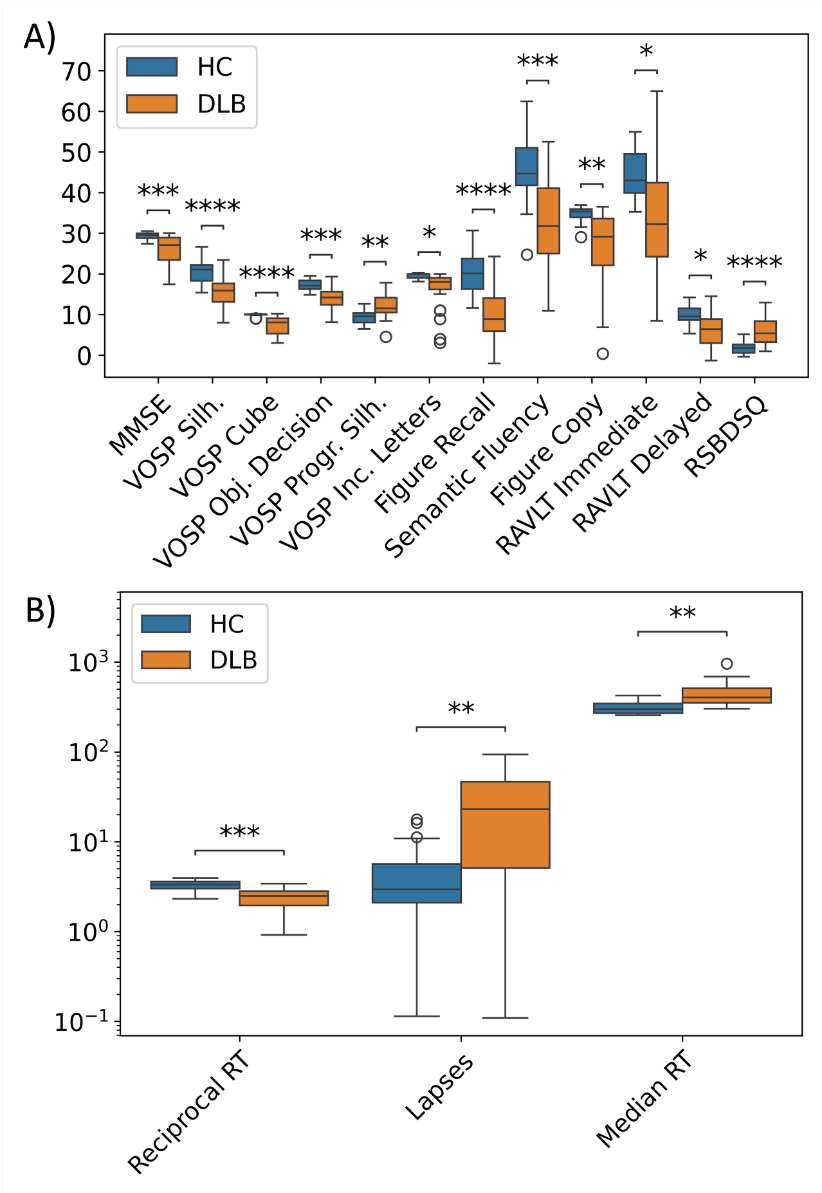


**Supplementary Figure 2. Significant differences in neuropsychological tests and PVT performance in DLB patients and HC.** a) Boxplots of significantly altered neuropsychological tests between DLB patients and HC. b) PVT metrics performances (logarithmic scale). *0.01 < p < 0.05; **0.001 < p < 0.01; ***p < 0.001.

DLB = Dementia with Lewy Bodies; HC = Helthy Controls; PVT = Psychomotor Vigilance Task, MMSE = Mini-Mental State Examination; VOSP = Visual Object and Space Perception Battery; VOSP Silh. = VOSP Silhouettes; VOSP Cube = VOSP Cube Analysis; VOSP Obj. Decision = VOSP Object Decision; VOSP Progr. Silh. = VOSP Progressive Silhouettes; VOSP Inc. Letters = VOSP Incomplete Letters; Figure Recall = Rey-Osterrieth Complex Figure Test delayed recall; Semantic Fluency = Verbal Fluency Test; Figure Copy = Rey-Osterrieth Complex Figure Test direct copy; RAVLT Immediate = Rey Auditory Verbal Learning Test immediate recall; RAVLT Delayed = Rey Auditory Verbal Learning Test delayed recall; RSBDSQ = REM Sleep Behavior Disorder Screening Questionnaire; Reciprocal RT = Psychomotor Vigilance Task – Reciprocal Reaction Time; Lapses = Psychomotor Vigilance Task – Lapses; Median RT = Psychomotor Vigilance Task – Median Reaction Time.


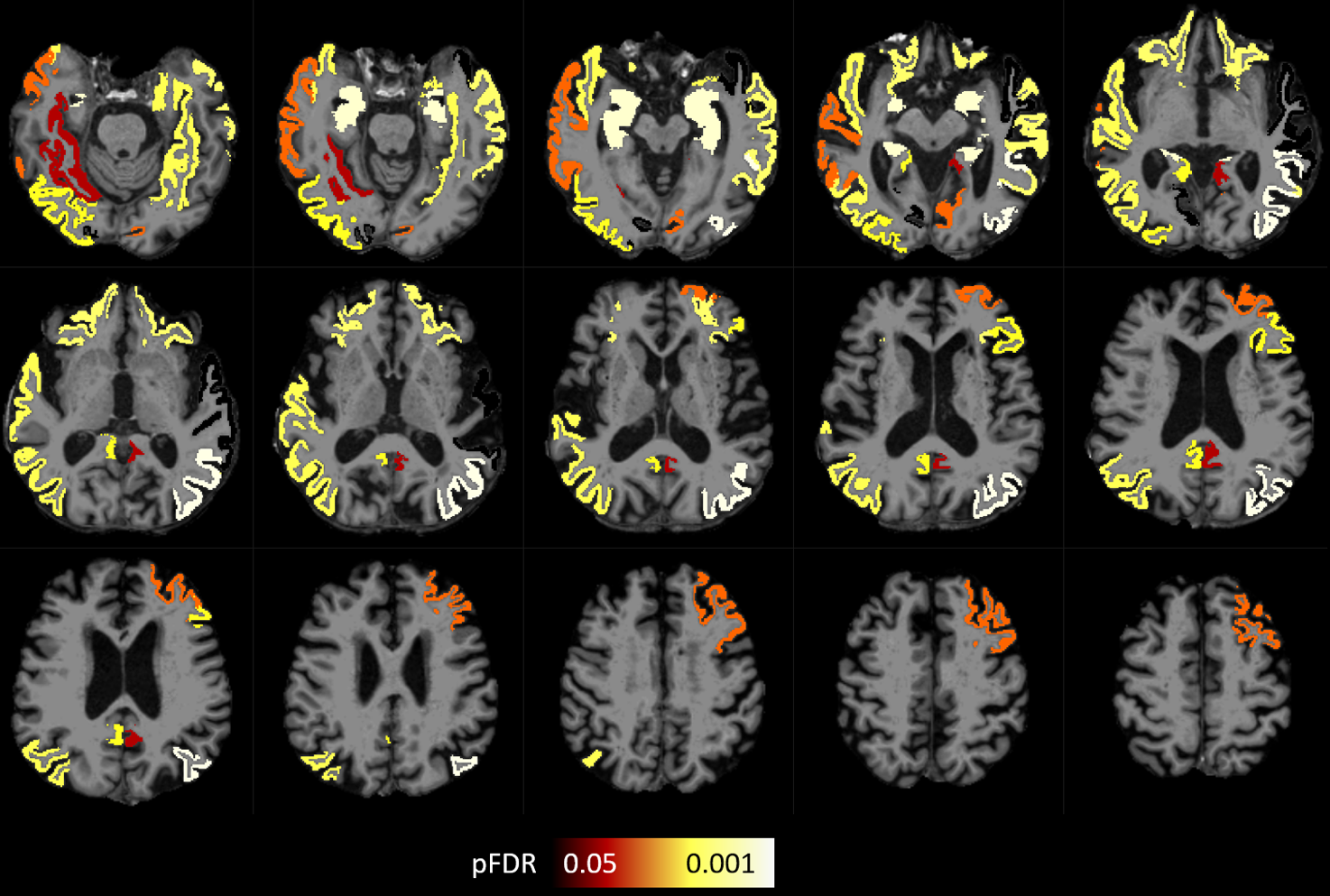


**Supplementary Figure 3. Widespread cortical and subcortical atrophy in DLB compared with HC.** Axial structural MRI slices from the Freesurfer segmentation with a significantly lower volume in DLB compared to HC. Colored overlays on an example brain indicate increasing levels of statistical significance, corrected for multiple comparisons using false discovery rate (pFDR < 0.05 to pFDR < 0.001). Widespread alterations emerged across all lobes, with the main involvement of the temporo-parietal areas and occipital ones, in addition to the subcortical nuclei. DLB = Dementia with Lewy Bodies; HC = healthy controls.
